# Supplementary material for: Crosstalk between Regnase-1 and -3 shapes mast cell survival and cytokine expression
Source: Life Sci Alliance. 2024 Jun 3;7(8):e202402784. doi: 10.26508/lsa.202402784 (PMC11147952; doi:10.26508/lsa.202402784)
Supplement: Supplementary file 15 [file LSA-2024-02784_TableS5.docx]

***Supplementary table 5. Materials used in this study***

| **REAGENT or RESOURCE** | **SOURCE** | **IDENTIFIER** |
| --- | --- | --- |
| Antibodies | | |
| anti-CD117 (c-Kit)-APC | Biolegend | Catalog #: 105812 |
| anti-CD117 (c-Kit)-APC/Cy7 | Biolegend | Catalog #: 105825 |
| anti-FcεRIα-PE | Biolegend | Catalog #: 134307 |
| anti-APC microbeads | Miltenyi Biotec | Catalog #: 130-090-855 |
| Mouse monoclonal Anti-Dinitrophenyl IgE | Sigma | Catalog #: D8406 |
| anti-TNF-α-PE/Cy7 | Biolegend | Catalog #: 506323 |
| anti-IL-6-PE | Biolegend | Catalog #: 504503 |
| anti-IL-6-APC | Biolegend | Catalog #: 504507 |
| anti-IL-13-PE | eBioscience | Catalog #: 12-7133-41 |
| anti-IL-13-eFluor 450 | eBioscience | Catalog #: 48-7133-80 |
| Anti-human TNF-α-PE | Biolegend | Catalog #: 502908 |
| Mouse monoclonal anti-Regnase-1 | R&D systems | Catalog #: MAB7875 |
| Rat anti-Regnase-1 (clone 15D11) | In-house production (Vigo Heissmeyer’s lab) | N/A |
| Rabbit polyclonal anti-Regnase-3 | GeneTex | Catalog #: GTX85195 |
| Rat anti-Regnase-3 (clone 4D3) | Helmholtz Munich Monoclonal Antibody Core Facility | Catalog #: MA10 |
| Mouse monoclonal anti-Beta Tubulin | Proteintech | Catalog #: 66240-1-Ig |
| Purified anti-HA.11 Epitope Tag antibody | Biolegend | Catalog #: 901501 |
| Monoclonal ANTI-FLAG® M2 antibody | Sigma | Catalog #: F1804 |
| anti-BrdU-Alexa Fluor 647 | BD Biosciences | Catalog #: 560209 |
| anti-rabbit IgG (H+L) Alexa Fluor 647 | Sigma | Catalog #: SAB4600393 |
| anti-mouse IgG (H+L) Alexa Fluor 647 | Thermo Fisher Scientific | Catalog #: A21236 |
| anti-mouse IgG (H+L) Alexa Fluor 594 | Thermo Fisher Scientific | Catalog #: A11005 |
| anti-mouse IgG (H+L) Alexa Fluor 568 | Thermo Fisher Scientific | Catalog #: A11004 |
| anti-rat IgG (H+L) Alexa Fluor 647 | Thermo Fisher Scientific | Catalog #: A21247 |
| anti-mouse IgG (H+L), Human ads-HRP | SouthernBiotech | Catalog #: 1031-05 |
| anti-rat IgG, HRP-linked | Cell Signaling Technology | Catalog #: 7077 |
| anti-rabbit IgG (whole molecule)-HRP | Sigma | Catalog #: A0545 |
| Normal mouse IgG | Sigma | Catalog #: 12-371 |
| Rat IgG isotype control | Thermo Fisher Scientific | Catalog #: 10700 |
|  |  |  |
| Chemicals, peptides, and recombinant proteins | | |
| Iscove's Modified Dulbecco's Medium (IMDM) | Gibco | Catalog #: 31980022 |
| Dulbecco's Modified Eagle Medium (DMEM) | Gibco | Catalog #: 61965059 |
| Fetal bovine serum | Gibco | Catalog #: 10270106 |
| GlutaMAX supplement | Gibco | Catalog #: 35050038 |
| MEM Non-Essential Amino Acids | Gibco | Catalog #: 11140068 |
| Penicillin-Streptomycin | Gibco | Catalog #: 15070063 |
| Sodium pyruvate | Gibco | Catalog #: 11360088 |
| β-mercaptoethanol | Gibco | Catalog #: 31350010 |
| Recombinant mouse IL-3 | In-house production | N/A |
| Recombinant Murine SCF | Peprotech | Catalog #: 250-03 |
| Albumin, dinitrophenyl (DNP) | Sigma | Catalog #: A6661 |
| Phorbol 12-myristate 13-acetate (PMA) | Sigma | Catalog #: P1585 |
| Ionomycin calcium salt | Sigma | Catalog #: I0634 |
| Recombinant Mouse IL-33 (carrier-free) | Biolegend | Catalog #: 580502 |
| Ultrapure LPS, E. coli 0111:B4 | Invivogen | Catalog #: TLRL3PELPS |
| Brefeldin A | Sigma | Catalog #: B6542 |
| TRI Reagent RT | Molecular Research Center, Inc. | Catalog #: RT111 |
| Protease inhibitor cocktail | Sigma | Catalog #: P8340 |
| PhosSTOP | Sigma | Catalog #: 4906845001 |
| siGLO Green Transfection Indicator control | Horizon Discovery/Dharmacon | Catalog #: D-001630-01-05 |
| Recombinant Cas9-NLS | In-house production | N/A |
| True Cut Cas9 Protein v2 | Thermo Fisher Scientific | Catalog #: A36499 |
| Alt-R CRISPR-Cas9 tracrRNA | Integrated DNA Technologies | Catalog #: 1072532 |
| Alt-R CRISPR-Cas9 tracrRNA, ATTO 550 | Integrated DNA Technologies | Catalog #: 1075927 |
| Nuclease-free duplex buffer | Integrated DNA Technologies | Catalog #: 11-01-03-01 |
| Alt-R CRISPR-Cas9 Electroporation Enhancer | Integrated DNA Technologies | Catalog #: 1075915 |
| SpeI-HF® | New England BioLabs Inc | Catalog #: R3133S |
| Pseudo-UTP | Jena Bioscience | Catalog #: NU-1139S |
| RNasin Plus RNase Inhibitor | Promega | Catalog #: N2615 |
| Actinomycin D from Streptomyces sp. | Sigma | Catalog #: A1410 |
| Formaldehyde | Sigma | Catalog #: F8775 |
| 4-nitrophenyl N-acetyl-β-D-glucosaminide | Sigma | Catalog #: N9376-250MG |
| Triton X-100 | Sigma | Catalog #: X100 |
| Glycine | Sigma | Catalog #: G7126 |
| Polyethylenimine (PEI) | Polysciences Inc | Catalog #: 23966 |
| PEG-8000 | Promega | Catalog #: V3011 |
| Dynabeads™ Protein G | Thermo Fisher Scientific | Catalog #: 10003D |
| Lysyl Endopeptidase®, Mass Spectrometry Grade (Lys-C) | FUJIFILM Wako Chemicals | Catalog #: 125-05061 |
| Sequencing Grade Modified Trypsin | Promega | Catalog #: V5111 |
| VECTASHIELD ® Antifade Mounting Medium with DAPI | VectorLabs | Catalog #: H-1200-10 |
| RNase A | Zymo Research | Catalog #: E-1008-30 |
| RNase I | Thermo Fisher Scientific | Catalog #: EN0601 |
|  |  |  |
| Critical commercial kits | | |
| Direct-zol RNA Microprep kit | Zymo | Catalog #: R2062 |
| qScript™ cDNA SuperMix | Quantabio | Catalog #: 733-1177 |
| PerfeCTa SYBR Green FastMix | Quantabio | Catalog #: 95055-500 |
| QuikChange II XL Site-Directed Mutagenesis Kit | Agilent | Catalog #: 200521 |
| Pierce™ BCA Protein Assay Kit | Thermo Fisher Scientific | Catalog #: 23227 |
| Clarity Western ECL Substrate | Bio-Rad | Catalog #: 1705060 |
| LIVE/DEAD™ Fixable Aqua Dead Cell Stain | Thermo Fisher Scientific | Catalog #: L34957 |
| LIVE/DEAD™ Fixable Blue Dead Cell Stain | Thermo Fisher Scientific | Catalog #: L23105 |
| eBioscience™ Foxp3 / Transcription Factor Staining Buffer Set | Thermo Fisher Scientific | Catalog #: 00-5523-00 |
| Neon™ Transfection System 10 µL Kit | Thermo Fisher Scientific | Catalog #: MPK1025 |
| Neon™ Transfection System 100 µL Kit | Thermo Fisher Scientific | Catalog #: MPK10025 |
| Qubit™ RNA High Sensitivity assay kit | Thermo Fisher Scientific | Catalog #: Q32852 |
| nCounter® Myeloid Innate Immunity Panel v2 | NanoString Technologies | Catalog #: XT-CSO-MMII2-12 |
| NucleoSpin Gel and PCR Clean‑up | Macherey-Nagel | Catalog #: 740609.50 |
| HiScribe™ T7 ARCA mRNA Kit (with tailing) | New England BioLabs Inc. | Catalog #: E2060S |
| Monarch RNA Cleanup Kit | New England BioLabs Inc. | Catalog #: T2040S |
| Dual-Luciferase® Reporter Assay System | Promega | Catalog #: E1910 |
| PE Annexin V Apoptosis Detection Kit I | BD Biosciences | Catalog #: 559763 |
| APC BrdU Flow Kit | BD Biosciences | Catalog #: 552598 |
| ReproSil-Pur 120 C18-AQ, 1.9 µm | Dr. Maisch HPLC GmbH |  |
| TNF alpha Mouse ELISA Kit, High Sensitivity | Thermo Fisher Scientific | Catalog #: BMS607-2HS |
|  |  |  |
| Deposited data | | |
| RBP2GO RNA-Binding Proteins database | https://pubmed.ncbi.nlm.nih.gov/33196814/ | https://rbp2go.dkfz.de/ |
| Raw and analyzed BMMC RNA-seq data | https://pubmed.ncbi.nlm.nih.gov/33479210/ | GEO: GSE145612 and Supplementary Table 1 |
| Raw and analyzed BMMC ATAC-seq data | https://pubmed.ncbi.nlm.nih.gov/33479210/ | GEO: GSE145612 and Supplementary Table 1 |
| Analyzed BMMC microarray data in ImmGen Database | https://pubmed.ncbi.nlm.nih.gov/27135604/ | GEO: GSE37448, https://www.immgen.org/, and Supplementary Table 1 |
| Raw and analyzed human skin MC RNA-seq data | https://pubmed.ncbi.nlm.nih.gov/37798647/ | GEO: GSE196862 and Supplementary Table 1 |
| Raw and analyzed human peripheral blood-derived MC RNA-seq data | https://pubmed.ncbi.nlm.nih.gov/31653482/ | GEO: GSE125887 and Supplementary Table 1 |
| Raw and analyzed Nanostring data | This paper | GEO: GSE240095 and Supplementary Table 2 |
| Raw and analyzed Reg-1 and Reg-3 IP-Mass spectrometry data | This paper | ProteomeXchange/PRIDE: PXD051849 and Supplementary Table 3 |
| Raw and analyzed Reg-1 KO RNA-seq data | This paper | GEO: GSE240035 and Supplementary Table 4 |
|  |  |  |
| Experimental models: | | |
| HEK293T | ATCC | CRL-3216 |
| C57BL/6 mice | Charles River Laboratories Italia |  |
| *Zc3h12a^fl/fl^* mice | https://pubmed.ncbi.nlm.nih.gov/29263935/ |  |
| HMC-1.1 | https://pubmed.ncbi.nlm.nih.gov/12519307/ |  |
| HMC-1.2 | https://pubmed.ncbi.nlm.nih.gov/12519307/ |  |
|  |  |  |
| Oligonucleotides | | |
| Primer for *Zc3h12a* qPCR, Forward 5’- CTGTGAACTGGTTTCTGGAG -3’ | This paper | N/A |
| Primer for *Zc3h12a* qPCR, Reverse 5’- GCGTGAACACCAAGATCTTC -3’ | This paper | N/A |
| Primer for *Zc3h12b* qPCR, Forward 5’- CAAAGGCCATAAAGATATCACGG -3’ | This paper | N/A |
| Primer for *Zc3h12b* qPCR, Reverse 5’- CGATGGTGTGAAGACGAGAATC -3’ | This paper | N/A |
| Primer for *Zc3h12c* qPCR, Forward 5’- CAGACATGGCCCAAGTCTG -3’ | This paper | N/A |
| Primer for *Zc3h12c* qPCR, Reverse 5’- CCCTTTCTGGGTGGTAGTATTTG -3’ | This paper | N/A |
| Primer for *Zc3h12d* qPCR, Forward 5’- GGAGGAAAGAGCCATCAAGGT -3’ | This paper | N/A |
| Primer for *Zc3h12d* qPCR, Reverse 5’- CATCGTAGCAGACCACTCGC -3’ | This paper | N/A |
| Primer for *Tnf* qPCR, Forward 5’- CTTCTGTCTACTGAACTTCGGG -3’ | This paper | N/A |
| Primer for *Tnf* qPCR, Reverse 5’- CAGGCTTGTCACTCGAATTTTG -3’ | This paper | N/A |
| Primer for *Tbp* qPCR, Forward 5’- CTGGAATTGTACCGCAGCTT -3’ | This paper | N/A |
| Primer for *Tbp* qPCR, Reverse 5’- ATGATGACTGCAGCAAATCG -3’ | This paper | N/A |
| Primer for *Gapdh* qPCR, Forward 5’- GCCTTCCGTGTTCCTACC -3’ | This paper | N/A |
| Primer for *Gapdh* qPCR, Reverse 5’- CCTCAGTGTAGCCCAAGATG -3’ | This paper | N/A |
| siRNA 1 for *Zc3h12a* knockdown, 5’- GCAGAGATCCTGTCTTACA -3’ | Horizon Discovery/Dharmacon | Catalog #: M-052076-00-0005 (D-052076-01) |
| siRNA 2 for *Zc3h12a* knockdown, 5’- GAGCGAGGCCACACAGATA -3’ | Horizon Discovery/Dharmacon | Catalog #: M-052076-00-0005 (D-052076-02) |
| siRNA 3 for *Zc3h12a* knockdown, 5’- GAAATGTACGTATGGAATC -3’ | Horizon Discovery/Dharmacon | Catalog #: M-052076-00-0005 (D-052076-03) |
| siRNA 4 for *Zc3h12a* knockdown, 5’- GAAAGGGCTGGTGTATATA -3’ | Horizon Discovery/Dharmacon | Catalog #: M-052076-00-0005 (D-052076-04) |
| siRNA 1 for *Zc3h12c* knockdown, 5’- GCCCAAGTCTGGATAATTT -3’ | Horizon Discovery/Dharmacon | Catalog #: M-062639-01-0005 (D-062639-01) |
| siRNA 2 for *Zc3h12c* knockdown, 5’- GCACTTAAGTTAGGTTATT -3’ | Horizon Discovery/Dharmacon | Catalog #: M-062639-01-0005 (D-062639-02) |
| siRNA 3 for *Zc3h12c* knockdown, 5’- GCACTACACCTTTAAGCAA -3’ | Horizon Discovery/Dharmacon | Catalog #: M-062639-01-0005 (D-062639-03) |
| siRNA 4 for *Zc3h12c* knockdown, 5’- GGATATTATTCCATGTTGA -3’ | Horizon Discovery/Dharmacon | Catalog #: M-062639-01-0005 (D-062639-04) |
| crRNA 1 for *Zc3h12a* knockout, 5’- CACCACTCCGTCGGATTCGA -3’ | Integrated DNA Technologies | Catalog #: Mm.Cas9.ZC3H12A.1.AA |
| crRNA 2 for *Zc3h12a* knockout, 5’- TTGCTTCCGTCGATGACCAC -3’ | Integrated DNA Technologies | Catalog #: Mm.Cas9.ZC3H12A.1.AB |
| crRNA 3 for *Zc3h12a* knockout, 5’- GGACAGGCTTCGTTCCACAA -3’ | Integrated DNA Technologies | Catalog #: Mm.Cas9.ZC3H12A.1.AD |
| crRNA 1 for *Zc3h12c* knockout, 5’- GGTATTCTCGAACCACATCA -3’ | Integrated DNA Technologies | Catalog #: Mm.Cas9.ZC3H12C.1.AA |
| crRNA 2 for *Zc3h12c* knockout, 5’- AATGCGAGAAACCTCATCCC -3’ | Integrated DNA Technologies | Catalog #: Mm.Cas9.ZC3H12C.1.AC |
| crRNA 3 for *Zc3h12c* knockout, 5’- CAATGGATACAGTCAATTCG -3’ | Integrated DNA Technologies | Catalog #: Mm.Cas9.ZC3H12C.1.AB |
| Scrambled crRNA control, 5’- GGTTCTTGACTACCGTAATT -3’ | Integrated DNA Technologies | N/A |
|  |  |  |
| Recombinant DNA | | |
| pUC57 mini-ZsGreen-P2A | This paper (synthesized by Genscript) | N/A |
| pUC57 mini-ZsGreen-P2A-FLAG-HA-Regnase-1 WT | This paper | N/A |
| pUC57 mini-ZsGreen-P2A-FLAG-HA-Regnase-1 D141N | This paper | N/A |
| pUC57 mini-FLAG-HA-Regnase-3 WT | This paper | N/A |
| pUC57 mini-FLAG-HA-Regnase-3 D252N | This paper | N/A |
| pUC57 mini-ZsGreen-P2A-Regnase-1 WT (human)-FLAG-HA | This paper | N/A |
| pUC57 mini-ZsGreen-P2A-Regnase-1 D141N (human)-FLAG-HA | This paper | N/A |
| pCDNA3 Mammalian Expression vector | Thermo Fisher Scientific | Catalog #: V011312 |
| pCDNA3-Regnase-1 WT | This paper | N/A |
| pCDNA3-Regnase-1 D141N | This paper | N/A |
| pCDNA3-Regnase-3 WT | This paper | N/A |
| pCDNA3-FLAG-Regnase-3_full-length | This paper | N/A |
| pCDNA3-FLAG-Regnase-3_1-548 aa | This paper | N/A |
| pCDNA3-FLAG-Regnase-3_1-410 aa | This paper | N/A |
| pCDNA3-FLAG-Regnase-3_1-245 aa | This paper | N/A |
| pCDH-EF1α-T2A-copGFP | System Biosciences | Catalog #: CD521A-1 |
| pCDH-Regnase-3 WT-T2A-copGFP | This paper | N/A |
| pCDH-Regnase-3 D252N-T2A-copGFP | This paper | N/A |
| pmirGLO Dual-Luciferase miRNA Target Expression vector | Promega | Catalog #: E1330 |
| pmiRGLO-*Zc3h12a* 3’UTR | This paper | N/A |
| pmiRGLO-*Tnf* 3’UTR | https://pubmed.ncbi.nlm.nih.gov/37386028/ | Addgene plasmid 207127 |
| pScalps-EGFP-Cre recombinase | https://pubmed.ncbi.nlm.nih.gov/37386028/ | Addgene plasmid 207132 |
| pScalps-Puro | https://pubmed.ncbi.nlm.nih.gov/27160912/ | Addgene plasmid 99636 |
| pScalps-Regnase-1 D141N | This paper | N/A |
| pScalps-Regnase-3 D252N | This paper | N/A |
| pMD2.G | Didier Trono’s laboratory | Addgene plasmid 12259 |
| psPAX2 | Didier Trono’s laboratory | Addgene plasmid 12260 |
|  |  |  |
| Software and algorithms | | |
| Immunological Genome Project (ImmGen) database | https://pubmed.ncbi.nlm.nih.gov/27135604/ | https://www.immgen.org/ |
| Fiji ImageJ v1.53h | https://pubmed.ncbi.nlm.nih.gov/22743772/ | https://imagej.net/software/fiji/ |
| FlowJo™ v10.6.0 | FlowJo, LLC | https://www.flowjo.com/ |
| GraphPad Prism v9 | GraphPad Software | https://www.graphpad.com/ |
| nSolver™ Analysis v4 | NanoString Technologies | Catalog #:MAN-C0019-08 |
| R version 4.2.1 | R Core Team (2022) | [https://www.R-project.org/](https://www.r-project.org/) |
| fastqc v.0.11.9 | Babraham Bioinformatics | https://www.bioinformatics.babraham.ac.uk/projects/fastqc/ |
| RSeQC v.4.0.0 | https://pubmed.ncbi.nlm.nih.gov/22743226/ | https://rseqc.sourceforge.net/ |
| HiSat2 v.2.2.1 | https://pubmed.ncbi.nlm.nih.gov/25751142/ | http://daehwankimlab.github.io/hisat2/download/ |
| featureCounts v.2.0.1 | https://pubmed.ncbi.nlm.nih.gov/24227677/ | https://subread.sourceforge.net/ |
| removeBatchEffect function in R | https://pubmed.ncbi.nlm.nih.gov/25605792/ | https://rdrr.io/bioc/limma/man/removeBatchEffect.html |
| Omics Playground web-based platform | https://pubmed.ncbi.nlm.nih.gov/33575569/ | https://bigomics.ch/omics-playground/ |
| DAVID Bioinformatics Resources v2023q2 | https://pubmed.ncbi.nlm.nih.gov/35325185/  https://pubmed.ncbi.nlm.nih.gov/19131956/ | https://david.ncifcrf.gov/ |
| RStudio version 4.1 | RStudio Team (2020) | http://www.rstudio.com/ |
| DESeq2 package in R | https://pubmed.ncbi.nlm.nih.gov/25516281/ | https://bioconductor.org/packages/release/bioc/html/DESeq2.html |
| RBP2GO database | https://pubmed.ncbi.nlm.nih.gov/33196814/ | https://rbp2go.dkfz.de/ |
| PANTHER 17.0 | https://pubmed.ncbi.nlm.nih.gov/34717010/  https://pubmed.ncbi.nlm.nih.gov/30804569/ | https://www.pantherdb.org/ |
| IGV 2.16.0 | https://pubmed.ncbi.nlm.nih.gov/21221095/ | https://software.broadinstitute.org/software/igv/2.16.x |
| Xcalibur™ software | Thermo Fisher Scientific | Catalog #: OPTON-30965 |
| MaxQuant software v.1.6.7.0 | https://pubmed.ncbi.nlm.nih.gov/19029910/ | https://www.maxquant.org/ |
| Andromeda peptide search engine | https://pubmed.ncbi.nlm.nih.gov/21254760/ | https://www.maxquant.org/ |
| MaxLFQ algorithm | https://pubmed.ncbi.nlm.nih.gov/24942700/ | https://www.maxquant.org/ |
| Perseus software v.1.6.2.3 | https://pubmed.ncbi.nlm.nih.gov/27348712/ | https://maxquant.net/perseus/ |
| Leica Application Suite X | Leica Microsystems | https://www.leica-microsystems.com/products/microscope-software/p/leica-las-x-ls/ |
|  |  |  |
| Others | | |
| QuantStudio 3 Real-Time PCR System | Thermo Fisher Scientific | Catalog #: A28136 |
| Fusion FX7 EDGE Imaging System | Witec | Catalog #: 1511 0200 1 |
| BD FACSymphony™ A5 Cell Analyzer | BD Biosciences |  |
| BD LSRFortessa™ Cell Analyzer | BD Biosciences |  |
| BD FACSymphony™ S6 Cell Sorter | BD Biosciences |  |
| Neon™ Transfection System | Thermo Fisher Scientific | Catalog #: MPK5000 |
| Qubit™ 3 Fluorometer | Thermo Fisher Scientific | Catalog #: Q33216 |
| nCounter® SPRINT Profiler | NanoString Technologies |  |
| GloMax® Discover | Promega | Catalog #: GM3000 |
| Stratalinker® UV crosslinker 2400 | Stratagene | Catalog #: 400076 |
| DynaMag™-2 Magnet | Thermo Fisher Scientific | Catalog #: 12321D |
| EASY-nLC™ 1200 HPLC System | Thermo Fisher Scientific | Catalog #: LC140 |
| Q Exactive HF Mass Spectrometer | Thermo Fisher Scientific |  |
| Leica Stellaris SP8 Confocal Laser Scanning Microscope | Leica Microsystems |  |
|  |  |  |
